# Inactivation of Pseudomonas aeruginosa by zinc oxide nanoparticles in aqueous solution

**DOI:** 10.1186/2047-2994-4-S1-I6

**Published:** 2015-06-16

**Authors:** A Maleki, M Ahmadi Jebeli, E Kalantar, H Daraei, B Davari, M Safari

**Affiliations:** 1 Kurdistan Environmental Health Research Center, Kurdistan University of Medical Sciences, Sanandaj, Iran, Islamic Republic Of; 2 Department of Microbiology and Immunology, School of Medicine, Alborz University of Medical Sciences, Karaj, Iran, Islamic Republic Of; 3 Department of Medical Entomology, School of Medicine, Hamadan University of Medical Sciences, Hamadan, Iran, Islamic Republic Of

## Introduction

Since ZnO nanoparticles (ZnO-NPs) exhibit strong antibacterial activities on a broad spectrum of bacteria the aim of this study was to evaluate the antimicrobial activity of Zno-NPs against *Pseudomonas* *aeruginosa* as a model for gram-negative bacteria.

## Methods

The average size of Zno-NPs was 20 nm, as determined through scanning electron microscopy. Muller Hinton broth was used as a growing medium for *Pseudomonas* *aeruginosa*. Photocatalytic experiment was carried out in a laboratory-scale batch reactor with low pressure ultraviolet irradiation (380 nm). Different experimental parameters such as amount of Zno-NPs, contact time, inorganic and organic substances and pH on photocatalytic inactivation of *Pseudomonas* *aeruginosa cells* have been studied. An initial *Pseudomonas* *aeruginosa* concentration of 10^8^ CFU/mL was used for all experiments.

## Results

Result showed that, almost all the initial *Pseudomonas* *aeruginosa* cell (10^8^ CFU/ml) was inactivated in 60 min in the presence of 2 g/l ZnO-NPs. Photocatalytic inactivation of bacteria was found to follow first order kinetics. The initial pH of the water did not play an important role on the inactivation rate within a range of 6–8 pH units. The amount of photocatalyst also plays an important role in photocatalytic inactivation rate. As the result showed increasing the photocatalyst amount provided more rapid inactivation.

## Conclusion

Addition of some inorganic ions to the suspension affects the sensitivity of *Pseudomonas* *aeruginosa* and caused to retard the inactivation rates. Since the sensitivity of *Pseudomonas* *aeruginosa* to photocatalytic treatment was fairly good, it is therefore, recommended to use this nano-particle for water treatment.

## Disclosure of interest

None declared.

